# Outdoor air pollution as a risk factor for Alzheimer’s disease: A systematic review

**DOI:** 10.1002/alz.089538

**Published:** 2025-01-09

**Authors:** Gurneet Dhillon, Nehal Hassan, Sarah Wilson, Ríona Mc Ardle, Li Su, Sarah Slight

**Affiliations:** ^1^ Newcastle University, Newcastle Upon Tyne UK; ^2^ University of Cambridge, Cambridge UK; ^3^ University of Sheffield, Sheffield UK

## Abstract

**Background:**

Outdoor air pollution is a global issue which poses a significant health risk. Modern neuroimaging techniques have revealed the detrimental impact of air pollution on brain health, in particular the development and progression of neurodegenerative diseases such as Alzheimer’s disease (AD).^(1)^ We conducted a systematic review to evaluate the effects of long‐term (months to years) exposure to outdoor air pollutants on the development and progression of AD using neuroimaging data.

**Method:**

This review followed PRISMA guidelines and registered in PROSPERO (CRD42023482979). Four large databases (MEDLINE, Embase, Scopus, and CINAHL) were systematically searched using words relating to “air pollution”, “neuroimaging”, and “Alzheimer’s disease”. The population researched was kept broad to include all ages. There were no geographical limits applied, and so included all countries. Articles were exported to Endnote (Endnote X9.3.3, Clarivate US), where duplicate articles were removed. Remaining articles were uploaded to the Rayyan and screened for eligibility. The Newcastle Ottawa Scale was used to assess the quality of included papers. A narrative synthesis was conducted, which involved grouping papers that focused on the same neuroimaging outcome and comparing and contrasting between studies.

**Result:**

Our search yielded 397 results, after removing duplicated (n=172), articles were removed at the title (n=192), abstract (n=8), and full text (n=17) stages. Eight articles met our inclusion criteria and focused on changes to white matter (n= 5), cortical thickness (n= 6), and grey matter (n= 2). Specific air pollutants (e.g., PM_2.5_) were associated with white matter reductions, and PM_10_ and NO_2_ with reduced cortical thickness. However, higher exposure to NO_x_ and NO_2_ was linked to better performance in cognition tests. Exposure to PM_2.5_ was associated with reduced grey matter, with study participants showing greater cognitive impairment. Air pollution exposure was associated with brain structure changes which are commonly seen in AD‐related pathology.

**Conclusion:**

Our results highlighted significant associations between specific air pollutant exposure and changes in different brain structures. Future research is needed to further investigate the relationship between air pollution exposure and cognitive decline.

**References**:

Block ML, Calderón‐Garcidueñas L. Air pollution: mechanisms of neuroinflammation and CNS disease. Trends Neurosci. 2009;32(9):506‐16.

